# The Antifungal Activity of Lactoferrin and Its Derived Peptides: Mechanisms of Action and Synergy with Drugs against Fungal Pathogens

**DOI:** 10.3389/fmicb.2017.00002

**Published:** 2017-01-18

**Authors:** Kenya E. Fernandes, Dee A. Carter

**Affiliations:** School of Life and Environmental Sciences, University of SydneySydney, NSW, Australia

**Keywords:** lactoferrin, lactoferricin, lactoferrampin, Lf(1–11), antimicrobial peptides, natural products, fungi, synergy

## Abstract

Lactoferrin is a multifunctional iron-binding glycoprotein belonging to the transferrin family. It is found abundantly in milk and is present as a major protein in human exocrine secretions where it plays a role in the innate immune response. Various antifungal functions of lactoferrin have been reported including a wide spectrum of activity across yeasts and molds and synergy with other antifungal drugs in combination therapy, and various modes of action have been proposed. Bioactive peptides derived from lactoferrin can also exhibit strong antifungal activity, with some surpassing the potency of the whole protein. This paper reviews current knowledge of the spectrum of activity, proposed mechanisms of action, and capacity for synergy of lactoferrin and its peptides, including the three most studied derivatives: lactoferricin, lactoferrampin, and Lf(1–11), as well as some lactoferrin-derived variants and modified peptides.

## Introduction

The rising incidence of severe fungal infections, due largely to increased numbers of immunocompromised patients, has made the discovery of new compounds that have effective antifungal activity or that support the activity of currently used antifungal agents more important than ever. Natural products are a significant source of novel antimicrobial compounds, and an increasing number are being identified from mammalian sources including milk proteins. One example is the multifunctional iron-binding glycoprotein, lactoferrin (Lf), and its various derived peptides. This article will focus on the spectrum of antifungal activity shown by Lf when used alone and in combination with other antifungal drugs, and will review currently proposed mechanisms of antifungal action for Lf and its derived peptides.

First identified almost 80 years ago in bovine milk (Sorensen and Sorensen, 1939) and later isolated and purified from humans and cows in 1960 (Groves, 1960), Lf is produced by various mammals including pigs, goats, dogs, mice, and horses (Lönnerdal and Iyer, 1995; Garcia-Montoya et al., 2012). In humans, it is a major protein secreted by the epithelial cells into exocrine fluids such as tears, saliva, vaginal fluids, gastrointestinal fluids, bile, urine, and sweat (Masson et al., 1968; van der Strate et al., 2001; González-Chávez et al., 2009; Park et al., 2011) but is present at the highest concentrations in human colostrum and breast milk (Lönnerdal and Iyer, 1995). Lf is involved in various physiological functions including iron regulation and the innate immune response, and has been demonstrated to have anti-inflammatory, anti-oxidant, anti-allergic, and anti-cancer properties (Conneely, 2001; Kruzel et al., 2006; Actor et al., 2009; Iigo et al., 2009; Burrow et al., 2011; Ogasawara et al., 2014) in addition to antimicrobial activity against a wide range of fungal, bacterial, viral, and parasitic pathogens (Jenssen and Hancock, 2009; Leboffe et al., 2009; Berlutti et al., 2011; Giansanti et al., 2013). The ability to bind and sequester iron is an important component of the antimicrobial activity of Lf (Jenssen and Hancock, 2009).

In addition to being an active intact protein, Lf is a rich source of antimicrobial peptides that are cleaved from the polypeptide chain by various proteolytic enzymes. As many of these enzymes are present in various body sites in humans, it is expected that a number of the cleaved peptides contribute to the normal functions of Lf in the body (Sinha et al., 2013). An increasing number of Lf-derived peptides are also being generated and modified by chemical synthesis (van der Kraan et al., 2005a). Many Lf peptides exhibit a broad spectrum of antimicrobial activity, have various different structural elements and modes of action, and appear to perform numerous functions including modulation of the inflammatory response, and stimulation of apoptosis (Farnaud et al., 2004; Sánchez-Gómez et al., 2008; van der Does et al., 2012).

## Structure and antifungal activity of whole lactoferrin

The structure of Lf consists of a single polypeptide chain containing ~700 amino acids folded into two globular lobes. These lobes are highly homologous to each other and are connected by an α-helical residue providing flexibility to the Lf molecule. Each lobe consists of two domains forming a single iron binding site, allowing each Lf molecule to strongly bind two ferric ions (Anderson et al., 1989). The open, iron-free form of Lf is known as apo-Lf while the closed, iron-rich form is holo-Lf, which differs in tertiary structure and is more resistant to proteolysis than apo-Lf (Jameson et al., 1998). In addition to binding Fe^3+^, Lf has also been observed to bind a range of other compounds including lipopolysaccharides, heparin, DNA, and metal ions including Cu^2+^, Zn^2+^, and Mn^2+^ (Baker, 1994).

Lf is most commonly purified from human (hLf) or bovine (bLf) milk but it has also been obtained from the milk and colostrum of several other mammals. Recombinant Lf (rLf) is increasingly being generated from microbial expression systems and through transgenic plants and animals (Lönnerdal and Iyer, 1995). Lf molecules from across species are highly homologous but have varying antifungal activity owing to small differences in structure. Furthermore, the level of activity of Lf with similar purities can vary substantially between different producers, ranging from reasonably active to inactive in the same test organism. bLf in particular has well-documented activity against a range of human and plant fungal pathogens including yeasts and molds (Table 1). Most widely studied is its activity on members of the *Candida* species, with *Candida tropicalis, Candida krusei*, and *Candida albicans* exhibiting the highest level of susceptibility while *Candida glabrata* has the lowest (Nikawa et al., 1993; Xu et al., 1998).

**Table 1:** **Antifungal spectrum of activity of Lf and derived peptides**.

**Position in Lf**	**Peptide**	**Source**	**Test organism**	**Inhibitory conc.^‡^**	**References**
Whole protein	Lactoferrin	Bovine	*Aspergillus niger*	IC_50_ = 578 *μ*g/mL	Nikawa et al., 1993; Wakabayashi et al., 1998; Xu et al., 1998; Kuipers et al., 1999; Lahoz et al., 2008; Lai et al., 2016
			Candida albicans^*^	MIC = 200–>6400 *μ*g/mL	
			Candida glabrata^*^	MIC = >6000 *μ*g/mL	
			Candida guilliermondii^*^	MIC not determined	
			Candida krusei^*^	IC_50_ = 20 *μ*g/mL	
			Candida parapsilosis^*^	MIC not determined	
			Candida tropicalis^*^	MIC not determined	
			Cryptococcus gattii^*^	MIC = 64 *μ*g/mL	
			Cryptococcus neoformans^*^	MIC = 64 *μ*g/mL	
			*Phoma exigua*	IC_50_ = 821 *μ*g/mL	
			*Rhizoctonia solani*	IC_50_ = 121 *μ*g/mL	
			*Saccharomyces cerevisiae*	MIC = 16 *μ*g/mL	
			*Sclerotium rolfsii*	IC_50_ = 231 *μ*g/mL	
			*Sclerotinia sclerotiorum*	IC_50_ = 31 *μ*g/mL	
			*Trichoderma viride*	IC_50_ = 952 *μ*g/mL	
1–11	Lf(1–11)	Human	*Candida albicans*	MIC not determined	Lupetti et al., 2000, 2008
			*Aspergillus fumigatus*	MIC = 4.3 *μ*M	
16–40	HLBD1	Human	*Candida albicans*	MMC = 6.2 *μ*g/ml	Kondori et al., 2011
17–26	Peptide 2	Bovine	Candida albicans^*^	MIC = 17.3–17.5 *μ*M	Ueta et al., 2001
17–30	bLf 17–30	Bovine	Candida albicans^*^	MIC = 5–10 *μ*M	van der Kraan et al., 2004
17–31	Lfcin B 17–31	Bovine	*Alternatria* sp.	MIC = 16 *μ*M	Muñoz and Marcos, 2006; Enrique et al., 2007
			*Aspergillus nidulans*	MIC = 4 *μ*M	
			*Botrytis cinerea*	MIC = 16 *μ*M	
			Dekkera bruxellensis^*^	MIC = 3.75–20 *μ*M	
			*Fusarium oxysporum*	MIC = 8 *μ*M	
			*Penicillium digitatum*	MIC = 4 *μ*M	
			*Penicillium expansum*	MIC = 8 *μ*M	
			*Penicillium italicum*	MIC = 4 *μ*M	
			*Pichia membranifaciens*	MIC not determined	
			*Saccharomyces cerevisiae*	MIC = 32 *μ*M	
			*Zygosaccharomyces bailii*	MIC = 8 *μ*M	
			*Zygosaccharomyces bisprous*	MIC = 12 *μ*M	
17–31	Lfcin H 17–31	Human	*Candida albicans*	MIC = 10 *μ*g/ml	Håversen et al., 2010
17–41	Lactoferricin	Bovine	Absidia corymbifera^*^	MIC = 40 *μ*g/ml	Bellamy et al., 1993, 1994; Wakabayashi et al., 1996, 1998, 1999
			*Aspergillus versicolor*	MIC = 10 *μ*g/ml	
			Candida albicans^*^	MIC = 0.8–400 *μ*g/ml	
			Candida glabrtata^*^	MIC = 80–120 *μ*g/ml	
			Candida guilliermondii^*^	MIC = 5–40 *μ*g/ml	
			Candida kefyr^*^	MIC = 2.5–10 *μ*g/ml	
			Candida krusei^*^	MIC = 10–20 *μ*g/ml	
			Candida parapsilosis^*^	MIC = 7.8–80 *μ*g/ml	
			Candida tropicalis^*^	MIC = 0.31–1.25 *μ*g/ml	
			Cladosporium trichoides^*^	MIC = 5 *μ*g/ml	
			*Cryptococcus albidus*	MIC = 24 *μ*g/ml	
			*Cryptococcus curvatus*	MIC = 9 *μ*g/ml	
			Cryptococcus neoformans^*^	MIC = 0.63 *μ*g/ml	
			*Cryptococcus uniguttulatus*	MIC = 6 *μ*g/ml	
			*Epidermophyton floccosum*	MIC = 0.31–2.5 *μ*g/ml	
			Exophiala dermatitidis^*^	MIC = 2 *μ*g/ml	
			*Fonsecaea pedrosoi*	MIC = 5 *μ*g/ml	
			Fusarium moniliforme^*^	MIC = 2.5–5 *μ*g/ml	
			*Microsporum canis*	MIC = 40 *μ*g/ml	
			*Microsporum gypseum*	MIC = 20–40 *μ*g/ml	
			*Nannizia gypsea*	MIC = 30 *μ*g/ml	
			*Nannizzia incurvata*	MIC = 18 *μ*g/ml	
			*Nannizzia otae*	MIC = 60 *μ*g/ml	
			Paracoccidioides brasiliensis^*^	MIC = 0.63–1.25 *μ*g/ml	
			*Penicillium pinophilum*	MIC = 45 *μ*g/ml	
			*Penicillium vermiculatum*	MIC = 45 *μ*g/ml	
			Phialophora verrucosa^*^	MIC = 5–10 *μ*g/ml	
			*Saccharomyces cerevisiae*	MIC = 0.63 *μ*g/ml	
			*Sporothrix schenckii*	MIC = 2–10 *μ*g/ml	
			Trichophyton mentagrophytes^*^	MIC = 6.3–45 *μ*g/ml	
			Trichophyton rubrum^*^	MIC = 13–60 *μ*g/ml	
			Trichophyton tonsurans^*^	MIC = 5–40 *μ*g/ml	
			Trichophyton violaceum^*^	MIC = 40 *μ*g/ml	
			Trichosporon cutaneum^*^	MIC = 1.25–18 *μ*g/ml	
18–31	Lfcin 18–31	Human	*Candida albicans*	MIC = 10 *μ*g/ml	Håversen et al., 2010
18–37	Lfcin B-20	Bovine	*Candida albicans*	MIC = 8 *μ*g/ml	Chen et al., 2006
18–37	Lfcin P-20	Porcine	*Candida albicans*	MIC = 32 *μ*g/ml	Chen et al., 2006
18–40	Lfpep	Human	*Candida albicans*	MIC = 18.7 *μ*M	Viejo-Diaz et al., 2005
			*Candida glabrata*	MIC = 9.3 *μ*M	
			*Candida guilliermondii*	MIC = 9.3 *μ*M	
			*Candida krusei*	MIC = 4.7 *μ*M	
			*Candida parapsilosis*	MIC = 9.3 *μ*M	
			*Candida tropicalis*	MIC = 9.3 *μ*M	
18–42	Lfcin B 18–42	Bovine	*Candida albicans*	MIC = 100 *μ*g/ml	Wakabayashi et al., 1999
			*Trichophyton mentagrophytes*	MIC = 12 *μ*g/ml	
19–31	Lfcin 19–31	Human	*Candida albicans*	MMC = 200 *μ*g/ml	Håversen et al., 2010
19–38	Lfcin H-20	Human	*Candida albicans*	MIC = 256 *μ*g/ml	Chen et al., 2006
20–25	Lfcin B 20–25	Bovine	*Saccharomyces cerevisiae*	MIC = >48 *μ*M	Muñoz and Marcos, 2006; Enrique et al., 2007
			*Dekkera bruxellensis*	MIC not determined	
			*Pichia membranifaciens*	MIC not determined	
			*Zygosaccharomyces bailii*	MIC not determined	
			*Zygosaccharomyces bisporus*	MIC not determined	
			*Cryptococcus albidus*	MIC not determined	
			*Penicillium digitatum*	MIC = 8 *μ*M	
			*Penicillium italicum*	MIC = 8 *μ*M	
			*Penicillium expansum*	MIC = 16 *μ*M	
			*Penicillium* sp.	MIC = 32 *μ*M	
			*Aspergillus nidulans*	MIC = 8 *μ*M	
			*Botrytis cinerea*	MIC = 16 *μ*M	
			*Fusarium oxysporum*	MIC = 16 *μ*M	
20–28	Lfcin B-9	Bovine	Candida albicans^*^	MIC = 25–32 *μ*g/ul	Wakabayashi et al., 1999; Chen et al., 2006
			*Trichophyton mentagrophytes*	MIC = 6 *μ*g/ul	
20–28	Lfcin P-9	Porcine	*Candida albicans*	MIC = 512 *μ*g/ml	Chen et al., 2006
20–31	HL10	Human	Candida albicans^*^	MMC = 100–200 *μ*g/ml	Håversen et al., 2010; Kondori et al., 2011
			Candida parapsilosis^*^	MMC = 100–200 *μ*g/ml	
			*Candida kefyr*	MMC = 100 *μ*g/ml	
			*Candida krusei*	MMC = 100 *μ*g/ml	
			Candida neoformans^*^	MMC = 50 *μ*g/ml	
21–26	Peptide 5	Bovine	Candida albicans^*^	MIC = 500 *μ*M	Ueta et al., 2001
21–29	Lfcin H-9	Human	*Candida albicans*	MIC = 256 *μ*g/ml	Chen et al., 2006
30–41	Peptide3	Bovine	Candida albicans^*^	MIC = 635 *μ*M	Ueta et al., 2001
152–182	Kaliocin-1	Human	*Candida albicans*	MIC = 150 *μ*M	Viejo-Diaz et al., 2005
			*Candida glabrata*	MIC = 150 *μ*M	
			*Candida guilliermondii*	MIC = 150 *μ*M	
			*Candida krusei*	MIC = 150 *μ*M	
			*Candida parapsilosis*	MIC = 150 *μ*M	
			*Candida tropicalis*	MIC = 150 *μ*M	
259–284	Lfampin 259–284	Bovine	*Candida albicans*	LC_50_ = 2.3 *μ*M	Bolscher et al., 2006
261–284	Lfampin 261–284	Bovine	*Candida albicans*	LC_50_ = 1.8 *μ*M	van der Kraan et al., 2005a
263–284	Lfampin 263–284	Bovine	*Candida albicans*	LC_50_ = 0.7 *μ*M	van der Kraan et al., 2005a
264–284	Lfampin 264–284	Bovine	*Candida albicans*	LC_50_ = 1.8 *μ*M	van der Kraan et al., 2005a
265–278	Lfampin 265–278	Bovine	*Candida albicans*	LC_50_ = >100 *μ*M	van der Kraan et al., 2005a
265–280	Lfampin 265–280	Bovine	*Candida albicans*	LC_50_ = 39 *μ*M	van der Kraan et al., 2005a
265–282	Lfampin 265–282	Bovine	*Candida albicans*	LC_50_ = 5.2 *μ*M	van der Kraan et al., 2005a
265–284	Lfampin 265–284	Bovine	*Candida albicans*	LC_50_ = 0.7 *μ*M	Bolscher et al., 2006
265–296	Lfampin 265–296	Bovine	*Candida albicans*	LC_50_ = 0.5 *μ*M	Bolscher et al., 2006
266–284	Lfampin 266–284	Bovine	*Candida albicans*	LC_50_ = 1.4 *μ*M	van der Kraan et al., 2005a
266–286	Cap-LFampinH-K	Human	*Candida albicans*	MIC not determined	Haney et al., 2009
268–284	Lactoferrampin	Bovine	*Candida albicans*	MIC = 4.3 *μ*g/ml	van der Kraan et al., 2005a
269–286	LFampinH-K	Human	*Candida albicans*	MIC not determined	Haney et al., 2009
270–284	Lfampin 270–284	Bovine	*Candida albicans*	LC_50_ = 26 *μ*M	van der Kraan et al., 2005a
Unknown	Hydrolysate	Human	*Penicillium* sp.	MIC = 60–300 *μ*g/mL	Liceaga-Gesualdo et al., 2001

* *Indicates multiple strains were tested within species*.

‡ *IC_50_, Inhibitory Concentration (50% Inhibition); LC_50_, Lethal Concentration (50% Mortality); MIC, Minimum Inhibitory Concentration; MMC, Minimum Microbiocidal Concentration. As different studies have used varying methods and sources of lactoferrin to determine inhibitory concentrations, these values may not be comparable between studies*.

Early studies with *Candida* species attributed the antifungal effect of Lf to its ability to sequester iron resulting in a fungistatic effect (Kirkpatrick et al., 1971), and inhibition of growth of *Candida* and *Cryptococcus* can be rescued by the addition of iron (Al-Sheikh, 2009; Lai et al., 2016). In *Aspergillus fumigatus*, the sequestration of Fe^3+^ by apo-Lf was shown to be important for host defense (Zarember et al., 2007) indicating that iron sequestration can play a role in the antifungal action of Lf *in vivo*. More recent studies now suggest that the main antifungal mechanism of Lf is iron-independent and occurs through a direct interaction of Lf with the fungal cell surface, leading to cell membrane damage and leakage. Supernatant protein assays and propidium iodide staining have shown that Lf alters cell surface permeability in *C. albicans, C. krusei*, and *Cryptococcus neoformans*, leading to cell death (Nikawa et al., 1993; Wakabayashi et al., 1996; Kondori et al., 2011). Alterations to the cell surface itself including protein leakage, the formation of surface blebs, swelling, and cell collapse have been seen in *Candida* isolates through scanning electron microscopy (Nikawa et al., 1993; Wakabayashi et al., 1996; Xu et al., 1998; van der Kraan et al., 2005b).

The cellular release of potassium ions, cytosolic acidification, changes in membrane potential, intracellular ROS accumulation, and chromatin condensation have all been detected in *C. albicans* following treatment with Lf indicating the induction of an apoptotic phenotype (Viejo-Díaz et al., 2004; Andres et al., 2008). The details by which Lf induces apoptosis-like processes in fungi remain largely unclear, however, recent studies have examined this mechanism in significant yeast species. In *Saccharomyces cerevisiae*, it was shown that hLf induces cell death in a mitochondrial and caspase-dependent way that is characterized by caspase activation, ROS accumulation and cytochrome c release (Acosta-Zaldivar et al., 2016). Work by the same group in *C. albicans* indicated that the proton-translocating ATPase Pma1p, which is a primary contributor to pH regulation in yeasts, was the target of hLf, inducing lethal mitochondrial dysfunction (Andres et al., 2016).

The combined use of Lf with other antifungal drugs has been increasingly studied in recent years. When combined with fluconazole, various sources of Lf have been observed to significantly enhance inhibitory activity and decrease the minimum inhibitory concentration (MIC) for several *Candida* species, including wild type and clinical strains, and ergosterol biosynthesis and azole-resistant mutants (Wakabayashi et al., 1998; Kuipers et al., 1999; Naidu et al., 2004; Venkatesh and Rong, 2008; Kobayashi et al., 2011). The mechanism of synergy between Lf and fluconazole is not yet well-understood and does not appear to result from an increased intracellular uptake of fluconazole, as the intracellular concentration of radiolabelled fluconazole was not affected by the addition of bLf (Kobayashi et al., 2011).

Several other azole drugs, including itraconazole, clotrimazole, and ketoconazole, as well as 5-fluorocytosine have been demonstrated to function synergistically with Lf in *C. albicans* (Wakabayashi et al., 1996; Kuipers et al., 1999). The polyene drugs amphotericin B and nystatin exhibited no interactions with bLf when tested in *Candida* species (Wakabayashi et al., 1996) although amphotericin B and bLf have been found to act synergistically in *Cryptococcus neoformans, Cryptococcus gattii*, and *S. cerevisiae*. The addition of exogenous iron did not rescue growth of *Cryptococcus* treated with amphotericin B and bLf, indicating that the mechanism of synergy is not solely due to iron chelation (Lai et al., 2016).

## Structure and antifungal activity of lactoferricin—the major peptide derived from lactoferrin

Lactoferricin (Lfcin) is found in the human gut as a natural breakdown product and was first generated by Bellamy et al. (1992) through pepsin hydrolysis of intact Lf. Bovine lactoferricin (bLfcin), comprising residues 17–41 from the N-terminal region of Lf, is simpler in structure but much more potent than human lactoferricin (hLfcin), which comprises residues 1–47. Both Lfcins possess an 18-residue loop region with a disulphide bridge and many positively charged and hydrophobic residues (Gifford et al., 2005).

The three dimensional structures of hLfcin and bLfcin differ from each other and from the homologous regions of the intact Lf protein, and it is thought these differences determine their different antifungal efficacies. While bLfcin forms a β-sheet confirmation in solution that contains a group of aligned hydrophobic residues well-suited to interactions with biological membranes, hLfcin forms a coiled structure in solution lacking the aligned residues and thus having weaker interactions with the target cell (Farnaud et al., 2004; Hunter et al., 2005; Alexander et al., 2012). Lfcin shows strongly enhanced antimicrobial activity compared to Lf and the antifungal efficacy of bLfcin, in particular, has been observed across a large range of various types of fungi (Table 1).

The primary antifungal mechanism of action of Lfcin appears to be through direct interaction with the fungal surface and disruption of the fungal membrane (Bellamy et al., 1993). Using confocal scanning laser microscopy, it was observed that bLfcin molecules were internalized by *C. albicans* cells within minutes (van der Kraan et al., 2005b) and that treatment with Lfcin resulted in dissipation of the proton gradient across the cell membrane (Gifford et al., 2005). In *C. albicans* and in the dermatophyte *Trichophyton mentagrophytes*, changes in ultrastructural features and an aggregation of cytoplasmic materials observed following treatment with Lfcin were traced to stimulation of ATP synthesis and extracellular secretion, resulting in pore formation in the plasma membrane (Bellamy et al., 1994; Ueta et al., 2001). Lfcin has also been shown to upregulate host defense via the induction of reactive oxygen species that stimulate the fungicidal activities of polymorphonuclear leukocytes (Kullberg et al., 1999). This ability to mount a combined assault on microbial pathogens is a common feature of natural host-derived products, most likely resulting from evolutionary pressures to maintain efficacy and prevent pathogens from developing resistance (Xu et al., 2009; Carter et al., 2016).

Synergy between bLfcin and azole antifungal drugs has been observed in *C. albicans*. In azole-resistant strains, the addition of low levels of bLfcin greatly increased inhibition by fluconazole and itraconazole and was seen to inhibit the growth of fungal hyphae (Wakabayashi et al., 1998). bLfcin has also been observed to function synergistically with clotrimazole and ketoconazole and it significantly reduced the formation of biofilms when used in combination with voriconazole and amphotericin B (Wakabayashi et al., 1996). The mechanistic basis of these synergistic actions remains unknown.

## Antifungal activity of other lactoferrin-derived peptides

### Lactoferrampin

Lactoferrampin (Lfampin), comprising residues 268–284 in the N1 domain of Lf, is located in close proximity to Lfcin in the tertiary structure of Lf. Identified as a molecule of interest due to its net positive charge and possession of a hydrophobic domain, Lfampin is thought to play a role in membrane-mediated activities of Lf (van der Kraan et al., 2004). Lfampin has demonstrated antifungal efficacy against *C. albicans* and is known to be more potent than Lf but has not yet been tested with a wide range of fungal organisms (Table 1). Similarly, whether Lfampin acts synergistically with antifungal drugs has not yet been determined.

Like Lfcin, the mechanism of action of Lfampin is by binding to and disrupting the cell membrane, and it was similarly observed to be internalized within a few minutes by *C. albicans*, resulting in membrane permeabilisation and the formation of vesicle-like structures (van der Kraan et al., 2005b). However, Lfampin differs greatly from Lfcin in amino acid composition, structure and orientation. In solution, the first 11 residues of bovine lactoferrampin (bLfampin) form an α-helical structure (Haney et al., 2007). A tryptophan residue in the N-terminal region has been shown to interact with model membranes and is contained in all varieties of Lfampin from different sources involved in membrane insertion indicating that it is responsible for this function (Haney et al., 2007; Sinha et al., 2013).

### Lf(1–11)

The Lf(1–11) peptide comprises the first 11 amino acid residues of the N-terminal region of Lf and exhibits antifungal activity against *C. albicans* and *A. fumigatus* (Table 1; Lupetti et al., 2000). The mechanism of action of Lf(1–11) also appears to rely on interactions with the fungal membrane, with important structural features including its highly cationic nature and the presence of hydrophobic valine (V6) and tryptophan (W8) residues (Bruni et al., 2016). Lupetti et al. (2000) demonstrated through residue substitution that the first two arginines at the N-terminus (R2, R3) are also necessary for the antifungal action of hLf(1–11) in *C. albicans*. hLf(1–11) appears to have immunomodulatory properties during *C. albicans* infection where it directs the differentiation of monocytes into dendritic cells that enhance the polarization of a Th17 response, which is an important component of the host defense against fungi (van der Does et al., 2012).

When combined with fluconazole, a synergistic antifungal effect has been seen across various *Candida* species including *C. glabrata, C. krusei, C. parapsilosis, C. tropicalis*, and fluconazole-resistant strains of *C. albicans*. At sub-inhibitory concentrations, these yeasts were seen to be killed when preincubated with hLf(1–11) before introducing fluconazole but not when fluconazole was introduced first, indicating that hLf(1–11) is responsible for initiating candidacidal activity while fluconazole acts synergistically during a later stage (Lupetti et al., 2003).

### Synthetic peptides based on lactoferrin

A wide variety of other derivatives of varying size and location have been generated from the whole Lf protein (Table 1). A number of these peptides were created in an attempt to facilitate the discovery of other antimicrobial sequences composed of short core residues within Lf. As an example of this, Chen et al. (2006) created and tested a range of short synthetic derivatives of human, bovine, and porcine Lfcins in an attempt to discover other antimicrobial sequences comprising core residues within Lf. This enabled them to find the antimicrobial domain in Lfcin of porcine origin, and to compare its potency to Lfcins of human and bovine origin.

Lf derivatives have also been designed to help clarify the relationship between structure and function of particular Lf-derived peptides. In one study, by testing a collection of peptides obtained by extending and/or truncating the C- or N- terminal ends of Lfampin, it was demonstrated that the highest level of antimicrobial activity corresponded with strongest membrane interactions and greatest ability to form an α-helix (Adão et al., 2011). In another study, a significant increase in candidacidal activity was seen in an extended form of Lfampin due to an increased positive charge near the C-terminal end (Haney et al., 2009). These studies demonstrate that there is capacity to optimize the activity of Lf-derived molecules by careful consideration of their sequence and structural properties.

### Modified and lactoferrin-like peptides

In recent years, an increasing number of studies have taken the path of modifying the original amino acid sequence of Lf peptides in order to develop more potent antifungal activity than in the parent peptides (Table 2). Various strategies designed to alter properties related to antifungal activity have been employed in the construction of these novel peptides such as modifying the length, net charge, and hydrophobicity of the peptide.

**Table 2:** **Antifungal spectrum of activity of modified Lf-derived or Lf-like peptides**.

**Peptide**	**Source**	**Modification**	**Amino acid sequence**	**Test organism**	**Inhibitory conc.^‡^**	**References**
HLR1r	Human	Derived from hLfcin; Arg-rich motif added to the C-terminal end to facilitate membrane interaction; N- and C-terminals capped.	Ac-FQWQRNMRKVRGSRRRRG-NH2	*Candida albicans*	MMC = 3 μg/mL	Björn et al., 2016
LBLP	*S. s. mutilans*	bLfcin-like peptide from the whole bodies of centipedes; found by BLASTx search (34.4% similarity).	RMKKLGNHKVSCERNTKRCRKAI	*Candida albicans*	MIC = 10 μM	Choi et al., 2013
				*Candida parapsilosis*	MIC = 10 μM	
				*Malassezia furfur*	MIC = 10–20 μM	
				*Trichosporon beigelii*	MIC = 20 μM	
L10	Bovine	Derived from bLf; residues 1–8 modified by selective homologous substitution of amino acids on the basis of hydrophobicity.	WFRKQLKW	Candida albicans^*^	MIC = 12.5–100 μg/mL	Mishra et al., 2013
				Candia tropicalis^*^	MIC = 12.5–100 μg/mL	
				*Candia krusei*	MIC = 25 μg/mL	
				*Candida glabrata*	MIC = 25 μg/mL	
				*Candida parapsilosis*	MIC = 6.25 μg/mL	
Lfchimera	Synthetic	Derived from bLfcin (17–30) and Lfampin; fusion (265–284) peptide generated synthetically.	FKCRRWQWRMKKLG-K-DLIWKLLSKAQEKFGKNKSR	*Candida albicans*	MIC not determined	Silva et al., 2013
LFT33	*E. coli*	Derived from bLfcin; fusion protein with thanatin; expressed recombinantly in *E. coli*.	FKCRRWQWRWKKLGAKPVPIIYCNRRTGKCQRM	*Candida albicans*	IC_50_ = 64 μg/mL	Feng et al., 2012
HLopt2	Human	Derived from hLf residues 20–31; modified by substitution with charged and hydrophobic amino acids.	CFQWKRAMRKVR	Candida albicans^*^	MMC = 12–25 μg/mL	Kondori et al., 2011
				Candida glabrata^*^	MMC = >400 μg/mL	
				Candida parapsilosis^*^	MMC = 12–25 μg/mL	
				*Candida krusei*	MMC = 12 μg/mL	
				*Candida kefyr*	MMC = 12 μg/mL	
				Cryptococcus neoformans^*^	MMC = 12 μg/mL	

* *Indicates multiple strains were tested within species*.

‡ *IC_50_, Inhibitory Concentration (50% Inhibition); MIC, Minimum Inhibitory Concentration; MMC, Minimum Microbiocidal Concentration*.

By the selective substitution of amino acids based on hydrophobicity in groups of residues derived from the N-terminal end of hLf, Kondori et al. (2011) and Mishra et al. (2013) created HLopt2 and L10, respectively. Björn et al. (2016) subsequently developed HLR1r from hLfcin by the addition of an Arg-rich motif designed to facilitate membrane interactions, together with capping of the N- and C- terminals to make them neutral. These three peptides demonstrated potent antifungal action against *Candida* species that was substantially greater than that of the corresponding residues from the native Lf peptide. Taking another approach, fusion peptides have been generated synthetically from the combination of multiple bioactive peptides: Lfchimera, a fusion of bLfcin and Lfampin, and LFT33, a fusion of bLfcin and the insect-derived antimicrobial peptide thanatin (Feng et al., 2012; Silva et al., 2013).

In addition to modifications of the natural peptide, the sequence of Lf has been used to search for other naturally occurring peptides with high homology in order to facilitate discovery of novel potential antifungals. The utility of this approach was demonstrated by the discovery of LBLP, a peptide with 34.4% similarity to bLfcin and produced from the bodies of centipedes, which was found to have strong antifungal activity against strains of *Candida, Malassezia*, and *Trichosporon* (Choi et al., 2013).

## Summary and conclusions

Lf and its major naturally cleaved peptide, Lfcin, have proven broad-spectrum antifungal action, with Lfcin exhibiting greater potency than the intact protein. The less well-characterized peptides including Lfampin and Lf(1–11) have also been seen to possess increased antifungal potency in *Candida* species but have not been tested extensively beyond this. Lf and Lfcin function synergistically primarily with azole antifungal drugs, however, knowledge of the spectrum and mechanism of these synergistic interactions is lacking, and synergy is largely unexplored for the other Lf-derived peptides.

Mechanistically, Lf and its peptides have been shown to function primarily through membrane destabilization, with immunomodulation and iron sequestration playing secondary roles. Although many aspects of Lf-derived peptides are quite conserved, differences in amino acid composition and tertiary structure contribute to their different functional properties. Novel alternative peptides that have been generated or modified from the original sequence of Lf provide insight into the relationship between structure and function. Lf and its derived peptides have great potential as leads for future antifungal drug development, however, considerable research is still required to understand the antifungal actions of these molecules as well as their potential to support current antifungals.

## Author contributions

This article was written by KF and was reviewed and edited by DC.

## Funding

Work on Lf in the Carter Lab has been supported by the Australian National Health and Medical Research Council (Grant Number APP1021267). KF is financially supported by an Australian Postgraduate Award.

### Conflict of interest statement

The authors declare that the research was conducted in the absence of any commercial or financial relationships that could be construed as a potential conflict of interest.
